# Gut microbiota impairs insulin clearance in obese mice

**DOI:** 10.1016/j.molmet.2020.101067

**Published:** 2020-08-26

**Authors:** Kevin P. Foley, Soumaya Zlitni, Brittany M. Duggan, Nicole G. Barra, Fernando F. Anhê, Joseph F. Cavallari, Brandyn D. Henriksbo, Cassandra Y. Chen, Michael Huang, Trevor C. Lau, Roxanne Plante, Michael Schwab, André Marette, Jonathan D. Schertzer

**Affiliations:** 1 Department of Biochemistry and Biomedical Sciences, Farncombe Family Digestive Health Research Institute McMaster University, Hamilton, Ontario, L8N 3Z5, Canada; 2 Departments of Genetics and Medicine, Stanford University, Stanford, CA, 94305, USA; 3 Quebec Heart and Lung Institute Research Center, Faculty of Medicine, Laval University, Quebec City, Quebec, G1V 4G5, Canada

**Keywords:** Diabetes, Glucose, Insulin, Microbiota, Obesity

## Abstract

**Objective:**

Hyperinsulinemia can be both a cause and consequence of obesity and insulin resistance. Hyperinsulinemia can result from increased insulin secretion and/or reduced insulin clearance. While many studies have focused on mechanisms triggering insulin secretion during obesity, the triggers for changes in insulin clearance during obesity are less defined. In this study, we investigated the role of the microbiota in regulating insulin clearance during diet-induced obesity.

**Methods:**

Blood glucose and insulin clearance were tested in conventional male mice treated with antibiotics and germ-free mice colonized with microbes from mice that were fed a control (chow) diet or an obesogenic high-fat diet (HFD). The composition of the fecal microbiota was analyzed using 16S rRNA sequencing.

**Results:**

Short-term HFD feeding and aging did not alter insulin clearance in the mice. Oral antibiotics mitigated impaired blood insulin clearance in the mice fed an HFD for 12 weeks or longer. Germ-free mice colonized with microbes from HFD-fed donor mice had impaired insulin but not C-peptide clearance. Microbe-transmissible insulin clearance impairment was only observed in germ-free mice after more than 6 weeks post-colonization upon HFD feeding. Five bacterial taxa predicted >90% of the variance in insulin clearance. Mechanistically, impaired insulin clearance was associated with lower levels of hepatic Ceacam-1 but increased liver and skeletal muscle insulin-degrading enzyme (IDE) activity.

**Conclusions:**

Gut microbes regulate insulin clearance during diet-induced obesity. A small cluster of microbes or their metabolites may be targeted for mitigating defects in insulin clearance and hyperinsulinemia during the progression of obesity and type 2 diabetes.

## Highlights

• Obesity impairs insulin clearance in mice, which is mitigated by antibiotics.
• The gut microbiota contributes to impaired insulin but not C-peptide clearance.
• The gut microbiota is a stand-alone factor that impairs insulin clearance.
• A cluster of related bacteria predict >90% of the variance in insulin clearance.
• Impaired insulin clearance is associated with lower hepatic Ceacam-1.

## Graphical abstract

**Figure:**
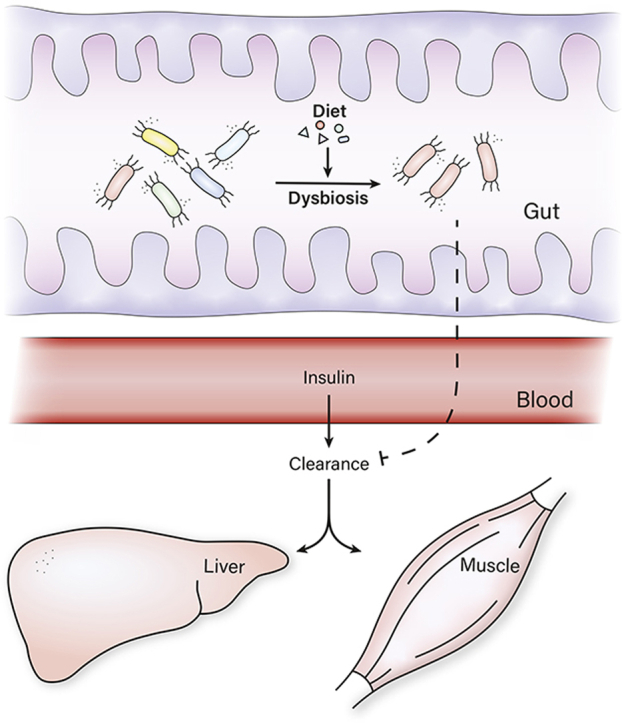

## 1 Introduction

Obesity is a predictor of insulin resistance and increased blood glucose and a risk factor for type 2 diabetes (T2D). Hyperinsulinemia has been implicated in the progression of obesity, insulin resistance, and T2D. Elevated insulin can be a cause and consequence of obesity and insulin resistance [[1], [2], [3]]. It is not yet clear how environmental factors, including gut-resident microbes, alter the relationship between hyperinsulinemia and obesity or insulin resistance. Dynamic insulin responses are controlled by insulin secretion versus insulin clearance followed by degradation. Increased insulin secretion and reduced (that is, impaired) insulin clearance can contribute to hyperinsulinemia. Obesity is associated with higher insulin secretion that can occur irrespective of changes in insulin sensitivity, whereas impaired insulin clearance is associated with insulin resistance during obesity [4].

Insulin secretion has been widely investigated in obesity and type 2 diabetes. Pancreatic beta cells sense blood glucose and secrete insulin, which promotes glucose uptake and lipogenesis and inhibits lipolysis and gluconeogenesis. Pancreatic beta cell characteristics and insulin secretion are modulated by neuronal and hormonal inputs, and defects in beta cell function underpin the risk of type 2 diabetes [[5], [6], [7]]. Insulin clearance, while less studied, is also modified by hormones such as incretins. For example, glucagon-like peptide-1 (GLP-1) administration in mice causes increased blood insulin levels in part by reducing insulin clearance [8]. Insulin clearance dynamics can be divided into hepatic and peripheral contributions. After insulin is secreted into the portal vein, insulin initially encounters the liver before accessing the general circulation. Approximately 50–80% of insulin may be depleted from the blood by hepatic uptake and degradation during first-pass insulin clearance [9,10]. Subsequently, skeletal muscle and the kidneys are key tissues that clear blood insulin via tissue-mediated insulin uptake and enzymatic degradation, which can protect against excessive insulin load and hypoglycemia [10].

Pancreatic-derived proinsulin is cleaved into two peptides: active insulin hormone and C-peptide. Measuring both blood insulin and C-peptide together can estimate the contributions of insulin secretion versus insulin clearance [11]. C-peptide is not subject to the same stringent clearance mechanisms of blood insulin, and it is possible to take advantage of this divergence in the mechanisms of hormone clearance to determine the specificity of insulin clearance versus the disappearance of co-secreted C-peptide or general clearance mechanisms for other peptides.

Insulin clearance is a key regulator of circulating insulin levels [12]. Hepatic insulin clearance is involved in the integrated response regulating insulin sensitivity, glucose production, and lipogenesis [12]. Impaired insulin clearance has been proposed as a contributor to (rather than a consequence of) insulin resistance [13]. Reduced insulin clearance may be driven by impaired hepatic or peripheral clearance, but it is not yet clear how obesity versus insulin resistance influences hepatic or peripheral insulin clearance [4,14]. In obese patients assessed for insulin resistance, the magnitude of lower insulin clearance coincided with a progressive increase in blood insulin levels [4]. Furthermore, reduced insulin clearance can occur prior to compensatory increases in insulin secretion, suggesting that reduced insulin clearance may be an early physiological response that is integrated into changes in insulin sensitivity [4]. Aging is associated with increased insulin levels and insulin resistance, but a comparison between mice aged 3 and 10 months suggested that hyperinsulinemia associated with this aging period is related to increased insulin secretion and not reduced insulin clearance [15].

Impaired insulin clearance triggers during obesity are ill-defined. Obesity is associated with metabolic endotoxemia, and lipopolysaccharides (LPS) derived from the cell wall of Gram-negative bacteria can impair insulin clearance [16,17]. Microbial pathogens such as *Salmonella typhimurium* lower insulin clearance and promote insulin resistance in mice [16]. Intestinal microbiota can regulate glucose metabolism [18,19] and insulin secretion [20]. For example, gut microbes can modulate insulin secretion in germ-free mice colonized with intestinal microbiota of various mouse strains [20]. We hypothesized that gut microbes also regulate insulin clearance, which could contribute to post-prandial hyperinsulinemia during diet-induced obesity. In this study, we define a role of intestinal microbiota in regulating insulin clearance during prolonged diet-induced obesity in mice. Colonization of germ-free mice with microbiota from chow-fed versus HFD-fed donor mice revealed that HFD-induced changes in a few related taxa could predict the microbe-induced influence on insulin clearance. We conclude that microbes from obese mice are contributors to impaired insulin clearance during diet-induced obesity, which may contribute to hyperinsulinemia, insulin resistance, and obesity.

## 2 Materials and methods

### 2.1 Mice

All of the procedures were approved by McMaster University's Animal Ethics Review Board (institutional approval number: AUP 20-01-03). Specific pathogen-free (SPF) *C5*7BL*/6J* mice were born at McMaster University. At 8–12 weeks of age, littermate mice were randomly placed on chow or 45% HFD diets. The control (chow) diet contained 17% calories from fat and ∼13% fiber content (Teklad 22/5 diet, catalog #8640), and the 45% HFD contained ∼6% fiber content, 45% calories derived from fat, and an energy density of 4.7 kcal per gram of food (Research Diets, D12451). When indicated, an antibiotic cocktail (1.0 mg/mL of ampicillin and 0.5 mg/mL of neomycin) was provided in their drinking water and changed every 2 days. Germ-free *C5*7BL*/6N* mice supplied by the Farncombe Gnotobiotic Unit of McMaster University were exported at 10–12 weeks of age and immediately colonized using soiled litter from the SPF *C5*7BL*/6J* donor mice. Colonization was re-enforced each day for the first week and once per week thereafter using new soiled litter from the SPF *C5*7BL*/6J* donor mice [19]. The mice were individually housed using ventilated racks and handled only in a level II biosafety hood [21]. Colonized, previously germ-free mice are referred to as recipient mice and were fed a chow diet upon export and maintained on the chow diet until they were switched to an HFD when indicated.

### 2.2 Insulin clearance

All of the metabolic tests were conducted after 6 h of fasting [22]. For insulin clearance during an oral glucose challenge, fasting blood glucose and blood samples (50 μL) were collected from the tail vein after 6 h of fasting. The mice were then given a 4 g/kg glucose dose by oral gavage and blood samples (50 μL) were collected from the tail vein at 10, 60, and 120 min post-gavage. For insulin clearance during an insulin challenge, the mice were given human insulin (1 U/kg, NovoRapid) or human C-peptide (50 μg/kg, Sigma) by intraperitoneal injection, and blood samples were collected from the tail vein sampling at 0, 5, 30, and 60 min post-injection. All of the blood samples were kept on ice after collection and then centrifuged at 10,000 g for 10 min at 4 °C. Plasma was collected into fresh tubes and stored at −80 °C. Mouse insulin and C-peptide were detected using a multiplex ELISA (Millipore) kit in the plasma samples collected during the oral glucose challenge. Human insulin (Mercodia) and human C-peptide (Millipore) were detected by ELISA kits in the plasma samples collected during the human insulin or human C-peptide challenges, respectively. To measure the insulin clearance after injection of human insulin, ELISA cross-reactivity with endogenous mouse insulin was assessed in t = 0 samples, which was the time point before the injection of human insulin. The insulin value obtained at t = 0 was subtracted from all of the subsequent measurements of plasma human insulin concentrations to assess the insulin clearance.

### 2.3 Expression of inflammatory and metabolic markers

Transcript levels of inflammatory and metabolic markers were assessed by qPCR as previously described [23]. Total RNA was obtained by phenol-chloroform extraction from ∼25–50 mg of indicated tissues. First-strand synthesis was conducted on ∼500 ng total RNA using SuperScript IV Reverse Transcriptase (Thermo Fisher Scientific). The transcript expression was measured using TaqMan assays with AmpliTaq Gold DNA polymerase (Thermo Fisher Scientific), and target genes were compared to the geometric mean of Rplp0 and 18S housekeeping genes using the ΔΔCT method.

### 2.4 Western blotting of phosphorylated and total Ceacam-1

Protein lysates were prepared from liver tissues of the Chow-R and HFD-R mice fed HFD. Phosphorylated carcinoembryonic antigen-related cell adhesion molecule (Ceacam-1) was assessed by immunoprecipitating total Ceacam-1 and blotting for phospho-tyrosine with a cocktail of monoclonal anti-phospho-tyrosine antibodies (rabbit no. 9411, 1:1000, Cell Signaling, and clone 4G10, 1:1000, Millipore, St. Louis, MO, USA). Phosphorylation of Ceacam-1 was expressed relative to the total immunoprecipitated Ceacam-1. Total Ceacam-1 was measured in protein lysates and expressed relative to immunoblotting with an actin loading control. When analyzing data across multiple gels, an additional loading control consisting of equal loading from all of the samples in that gel was used to normalize the protein expression.

### 2.5 Measurement of insulin-degrading enzyme (IDE) activity

IDE activity was assessed in the liver and muscle protein lysates using a SensoLyte 520 IDE Activity Assay Kit (catalog #AS-72231, AnaSpec, Fremont, CA, USA) according to the manufacturer's instructions. Activity was expressed as RFU/mg protein over a time course of 60 min, with sampling every 5 min. Reaction rates were calculated as the slope of the linear regression between 10 and 50 min.

### 2.6 Bacterial profiling

Fecal samples were collected and processed as previously described [19]. Briefly, DNA was purified using ZymoBIOMICS DNA kits (D4300, Zymo Research Corporation). Following mechanical disruption, we also conducted 2 enzymatic lysis steps consisting of lysis solution 1 (50 mg/mL of lysozyme and 20% RNase, Sigma R6148) at 37 °C for 1 h and lysis solution 2 (25 μL of 25% SDS, 25 μL of 5 M NaCl, and 50 μL of 10 mg per mL proteinase K) at 60 °C for 30 min. Illumina-compatible PCR amplification of the variable 3 (V3) region of the 16S rRNA gene was completed on each sample before sequencing on an Illumina MiSeq platform. A minimum of 26,000 reads per sample were acquired. Sequenced data were processed using a custom pipeline. Operational taxonomic units (OTUs) were grouped using Abundant OTU + based on a 97% similarity, and the 2013 version of the Greengenes reference database was used to assign taxonomy to the OTU Ribosomal Database Project (RDP) classifier in Quantitative Insights into Microbial Ecology (QIIME) [19]. QIIME and R scripts were used to generate plots of the taxonomy data to conduct statistical tests. Microbial taxonomy was expressed as relative abundance per sample. In heat maps, the relative abundance was expressed as log_2_ fold changes from the median of the entire cohort as described in each figure. All of the relative abundance values of 0 were assigned 1 × 10^−7^ in the heat maps, the lowest detectable decimal value in the relative abundance, to obtain the logarithmic transformation of the fold change. Statistical analyses were conducted using the relative abundance values. R packages used for the data analysis and visualization included vegan, ggplot2, tidyr, dplyr, ggtree, and corrplot.

Phylogenetic analysis of the 16S rRNA genomic sequences was conducted with QIIME 2 (Bolyen et al., 2019). For every consensus lineage (taxonomic classification) assigned using the QIIME 2 classifier, those that were present at 10 reads or more across all of the recipient mice (28 samples) were used for the analysis. For each of the consensus lineages, the amplicon sequence variant (ASV) with the highest total number of reads in the dataset was used as a representative of the taxon. A total of 112 sequences were aligned and used to construct a phylogeny using the QIIME 2 align-to-tree-mafft-fasttree command (Katoh et al., 2002, Price et al., 2010). The phylogenetic tree was edited using the R package ggtree and visualized using the Interactive Tree of Life (iTOL) [24].

### 2.7 Statistical analysis

To measure insulin or C-peptide during host metabolic tests, an unpaired two-tailed student's t-test was used to compare two groups and ANOVA and Tukey's post hoc analysis were used to compare more than two groups. Statistical significance was accepted at p < 0.05. Analysis and data visualization of microbial populations was conducted in R [25]. The variance in the microbiome was partitioned with an Adonis analysis of variance on Bray-Curtis dissimilarities calculated from the relative OTU abundances using the vegan package in R [26]. A pairwise Wilcoxon test was used for the non-parametric analysis of variance between groups with the significance threshold set to p < 0.05. Adjustment of the false discovery rate (FDR) was calculated with the Benjamini-Hochberg method, and statistical significance was accepted at p < 0.05 [27].

## 3 Results

### 3.1 High-fat feeding impaired insulin clearance during an oral glucose challenge in the mice

**Figure 1:**
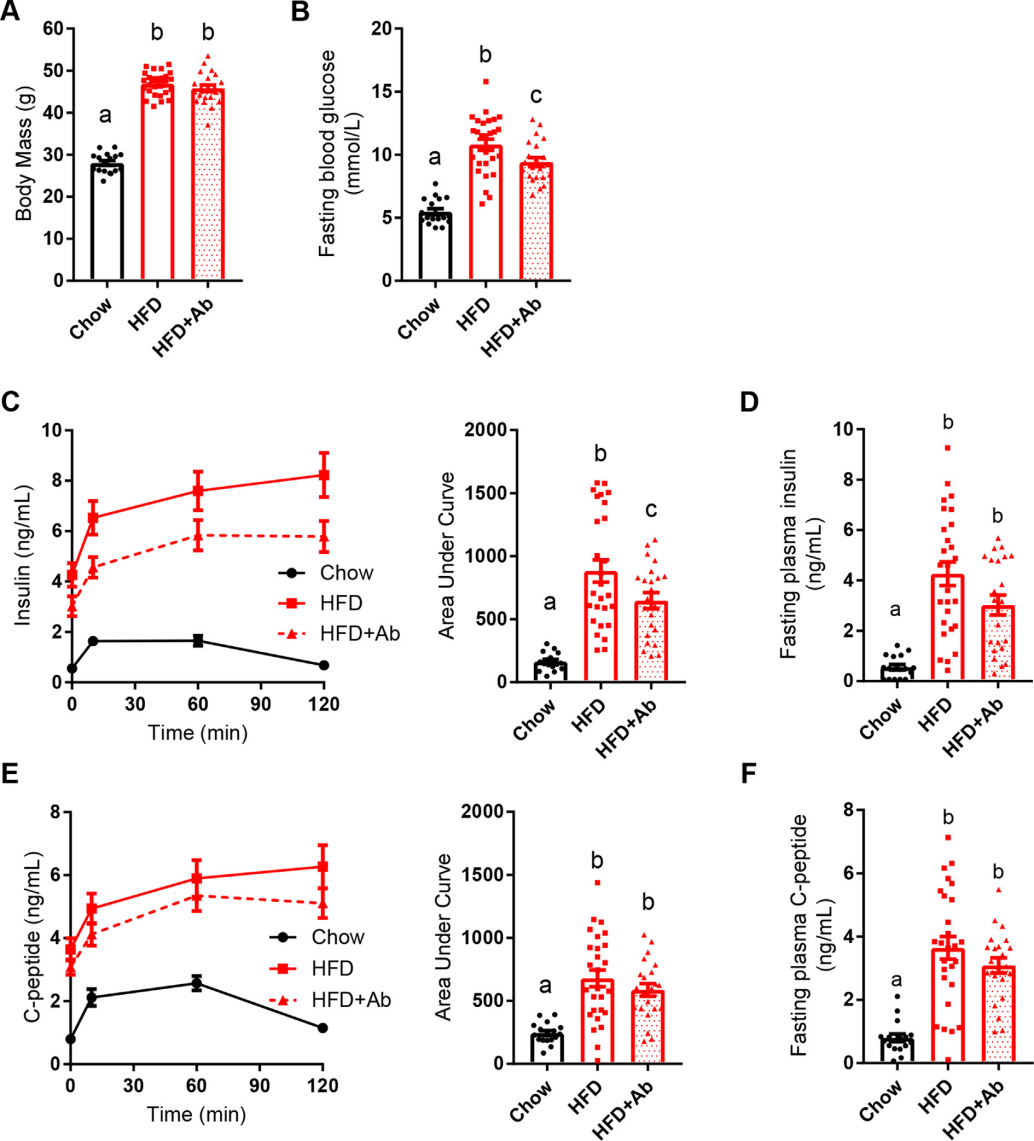
**Antibiotics mitigated impaired insulin clearance during an oral glucose challenge in obese mice.** Male mice were fed a control (chow) diet or an obesogenic low-fiber HFD for 14 weeks. A subset of the HFD-fed mice was given antibiotics (1 g/L of ampicillin and 0.5 g/L of neomycin) in their drinking water during the last 2 weeks (Chow = 17, HFD = 27, HFD + Ab = 22). **A)** Body mass and **B)** fasting blood glucose after week 14 of HFD feeding. **C-D)** Insulin and **E-F)** C-peptide were measured in plasma by ELISA and collected from each time point (0, 10, 60, and 120 min) during an oral glucose challenge (4 g/kg). **Data information:** All of the values are mean +/− SEM. Statistical significance was measured as p < 0.05 using one-way ANOVA. Post hoc analysis was conducted using Tukey's multiple comparisons test. Groups of mice denoted by different letters are statistically different from one another. Each dot/symbol indicates one mouse.

Intestinal microbiota can regulate blood glucose and insulin secretion [19,20], but it was unknown if gut microbes regulate insulin clearance. We fed the mice an obesogenic low-fiber HFD or control (chow) diet for 14 weeks. Some of the mice were supplemented with antibiotics (1 g/L of ampicillin and 0.5 g/L of neomycin) in the drinking water during the last 2 weeks of high-fat feeding. This antibiotic cocktail was chosen specifically for its ability to lower blood glucose and improve insulin sensitivity in HFD-fed mice without causing weight loss [21,28]. The mice fed an HFD had higher body masses and higher fasting blood glucose relative to the chow diet-fed mice (Figure 1A,B). As expected, treatment of the HFD-fed mice with antibiotics lowered fasting blood glucose (Figure 1B) but antibiotics did not alter body mass (Figure 1A). After 14 weeks on each diet, we conducted an oral glucose challenge (4 g/kg, p.o.) and collected blood samples to analyze the insulin and C-peptide plasma concentrations. The HFD-fed mice had higher fasting insulin and a greater increase in blood insulin concentrations during the oral glucose challenge relative to the chow-fed mice (Figure 1C,D). C-peptide was elevated both in the fasted state and during the oral glucose challenge in the HFD-fed mice compared to the chow diet-fed mice (Figure 1E,F). Antibiotic treatment attenuated the increase in blood insulin but not C-peptide during the oral glucose challenge (Figure 1C,E). Antibiotic treatment did not significantly lower fasting insulin or C-peptide levels in the serum (Figure 1D,F). However, antibiotic treatment in the HFD-fed mice produced a trend toward lowering fasting insulin in the HFD + Ab versus HFD mice (p = 0.0682) (Figure 1D). Therefore, we accounted for the basal insulin concentration and calculated the incremental area under the curve (AUC) for a change in plasma insulin during an oral glucose load. When correcting for baseline (that is, fasting insulin), our data showed that antibiotics did not alter the incremental changes in blood insulin (Supplemental Figure 1). These data prompted more direct testing of how gut microbes alter blood insulin clearance. Overall, these results suggest that antibiotics may improve (that is, increase) insulin clearance during an oral glucose load coincident with subtle changes in fasting insulin, but antibiotics did not alter C-peptide kinetics after an oral glucose challenge in the mice chronically fed an obesogenic HFD.

### 3.2 Antibiotics increased insulin clearance during long-term high-fat feeding in the mice

**Figure 2:**
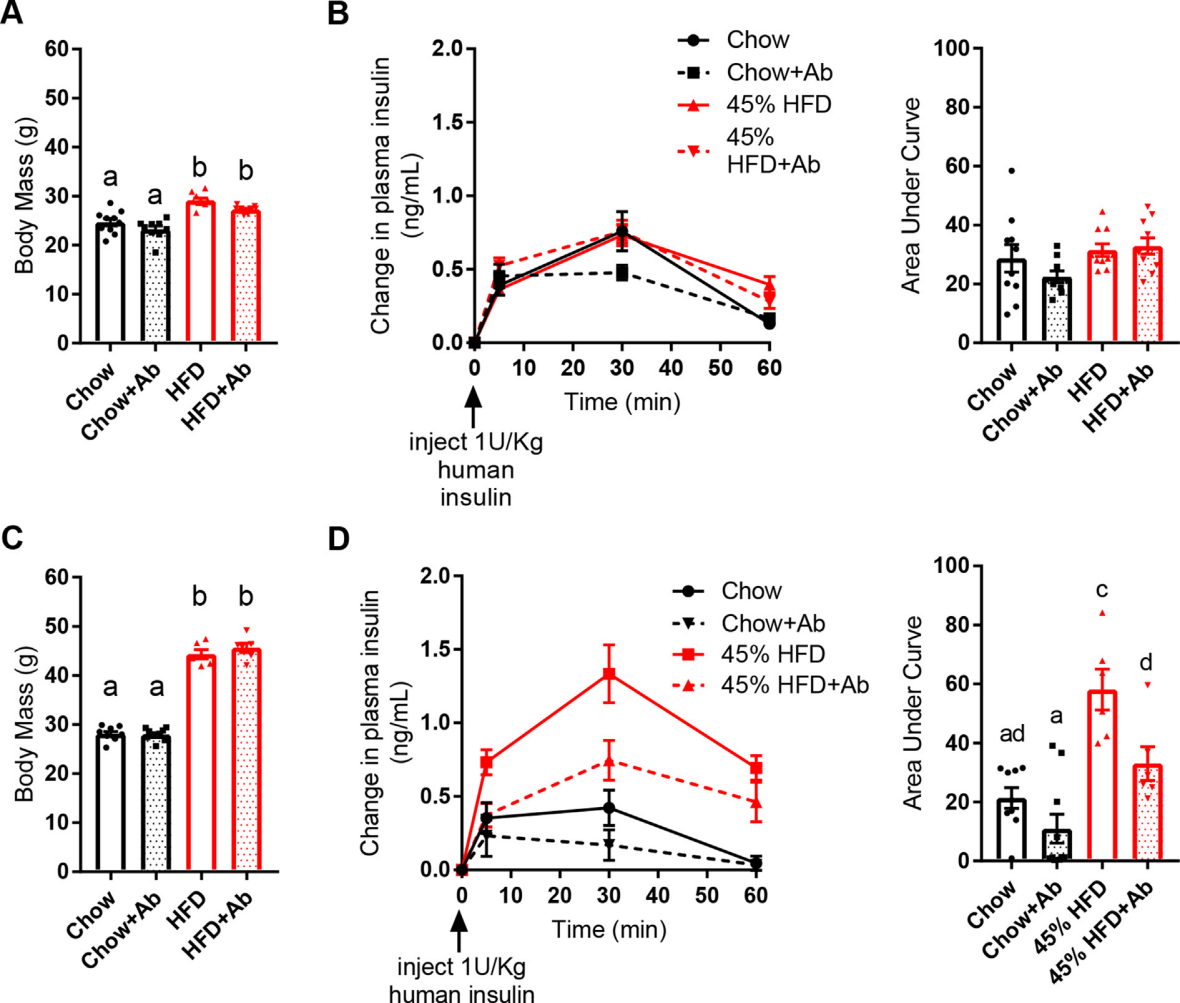
**Antibiotics mitigated impaired insulin clearance after chronic HFD feeding in obese mice. A)** Body mass and **B)** insulin clearance were measured in male mice fed a control chow diet or an obesogenic low-fiber HFD for 2 weeks +/− antibiotics (1 g/L of ampicillin and 0.5 g/L of neomycin) in their drinking water (N = 9–10 per group). The human insulin concentration was measured in plasma at indicated time points following injection of human insulin (1 U/kg, i.p.). **C)** Body mass and **D)** insulin clearance in male mice fed a chow diet or an HFD for 10 weeks followed by 2 additional weeks +/− antibiotics in their drinking water (N = 6–10 per group). **Data information:** All of the values are mean +/− SEM. Statistical significance was measured as p < 0.05 using two-way ANOVA and post hoc analysis using Tukey's multiple comparisons test. Groups denoted by different letters are statistically different from one another. Each dot/symbol indicates one mouse.

We then directly investigated insulin clearance independently of control mechanisms engaged by an oral glucose load. Human insulin was injected (1 U/kg, i.p.) in lean and obese mice and its presence in the circulation was assessed as a readout of insulin clearance. Two weeks of HFD feeding increased body mass (Figure 2A) but did not alter insulin clearance (Figure 2B). Oral antibiotics did not alter body mass or insulin clearance in the lean mice or mice fed an HFD for 2 weeks (Figure 2A,B). However, compared to the age-matched chow diet-fed mice, HFD feeding for 12 weeks increased body mass (Figure 2C) and impaired insulin clearance, which is characterized by the increased AUC in Figure 2D. Oral antibiotics during the last 2 weeks of HFD feeding improved insulin clearance without changing body mass (Figure 2C,D). Together, these data support a model in which the intestinal microbiota contributes to impaired insulin clearance during chronic diet-induced obesity.

### 3.3 Diet but not aging altered insulin clearance

**Figure 3:**
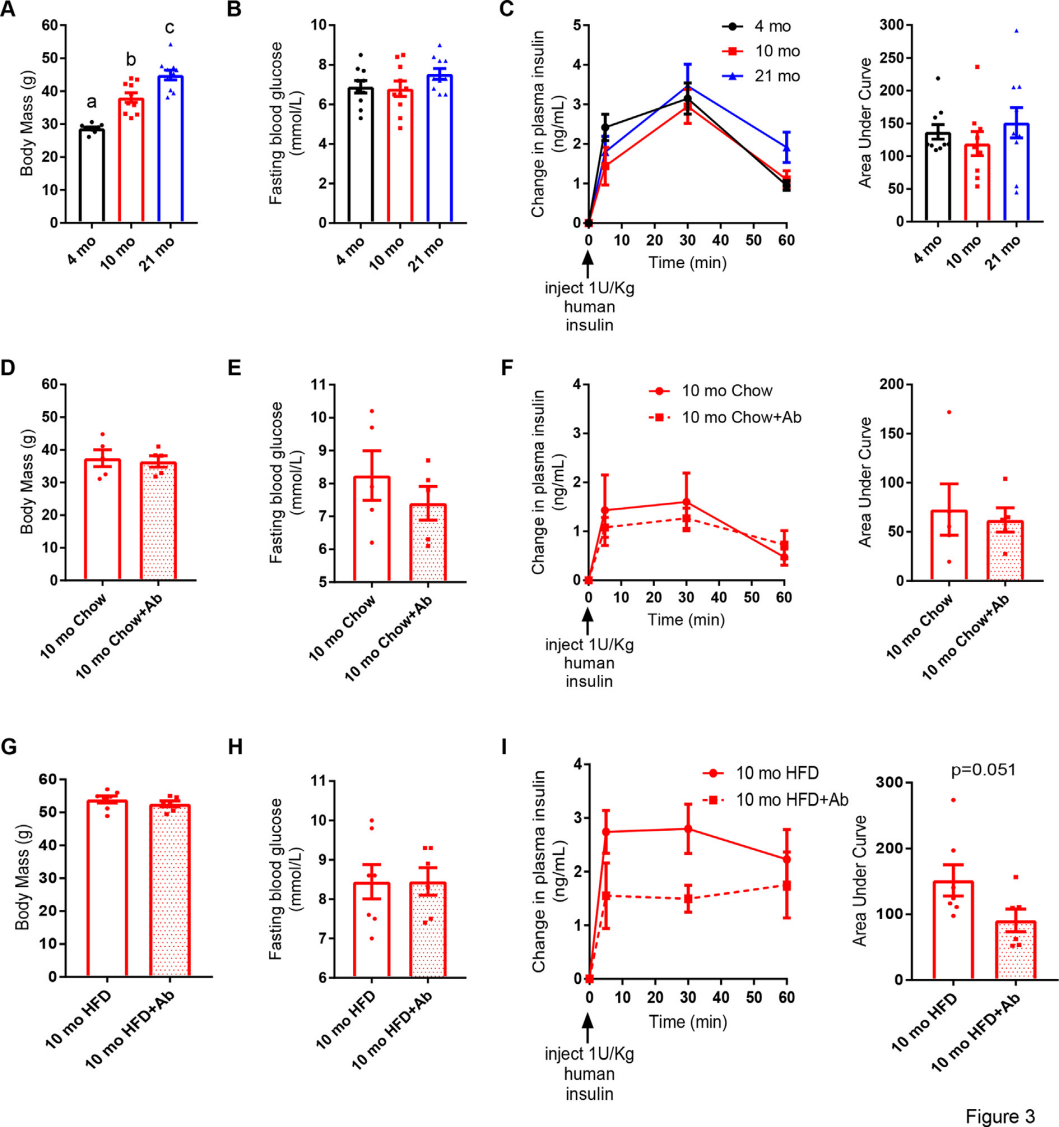
**Diet but not aging altered insulin clearance. A-C)** Male mice were fed a control chow diet for 4 months, 10 months, or 21 months (N = 10 per group). **A)** Body mass, **B)** fasting blood glucose, and **C)** insulin clearance after injection of human insulin (1 U/kg, i.p.). **D-F)** Male mice aged 10 months were fed a control chow diet +/− antibiotics (1 g/L of ampicillin and 0.5 g/L of neomycin) in their drinking water for 2 weeks (N = 5 per group). **D)** Body mass, **E)** fasting blood glucose, and **F)** insulin clearance after injection of human insulin (1 U/kg, i.p.). **G-I)**Male mice aged 10 months were fed an HFD for 35 weeks followed by 2 additional weeks +/− antibiotics in their drinking water (N = 6–7 per group). **G)** Body mass, **H)** fasting blood glucose, and **I)** insulin clearance after injection of human insulin (1 U/kg, i.p.). **Data information:** All of the values are mean +/− SEM. Statistical significance was measured as p < 0.05 using one-way ANOVA (A–C) or student's t-test (D–I). Post hoc analysis was conducted using Tukey's multiple comparisons test. Groups denoted by different letters are statistically different from one another. Each dot/symbol indicates one mouse.

Our data showed that antibiotics improved insulin clearance in the mice fed an HFD for a prolonged period (12 weeks) but not during short-term HFD feeding (2 weeks) without changes in body mass. Aging alters the composition of the gut microbiome and promotes inflammation and insulin resistance [29]. To investigate if aging contributes to defects in insulin clearance, we tested insulin clearance in the chow-fed mice that were 4, 10, or 21 months of age. Although body mass increased with age (Figure 3A), fasting blood glucose and insulin clearance were unaltered in the aged mice (Figure 3B,C). Thus, our data support a model in which defects in insulin clearance in the mice fed an HFD for 12 weeks or longer occurred independently of aging. These data are concordant with a previous report that demonstrated that aging did not alter insulin clearance in 3- and 10-month-old mice [15]. We next tested the effect of antibiotics on insulin clearance in the 10-month-old chow-fed or HFD-fed mice. Two weeks of oral antibiotics had no effect on body mass, fasting blood glucose, or insulin clearance in the 10-month-old chow-fed mice (Figure 3D–F). However, 2 weeks of oral antibiotics improved insulin clearance in the 10-month-old mice fed an HFD for 37 weeks without changes in body mass or fasting blood glucose (Figure 3G–I). These results strengthen the hypothesis that the intestinal microbiota causes impaired insulin clearance during prolonged diet-induced obesity.

### 3.4 The microbiota from the HFD-fed mice impaired insulin clearance without changes in obesity

**Figure 4:**
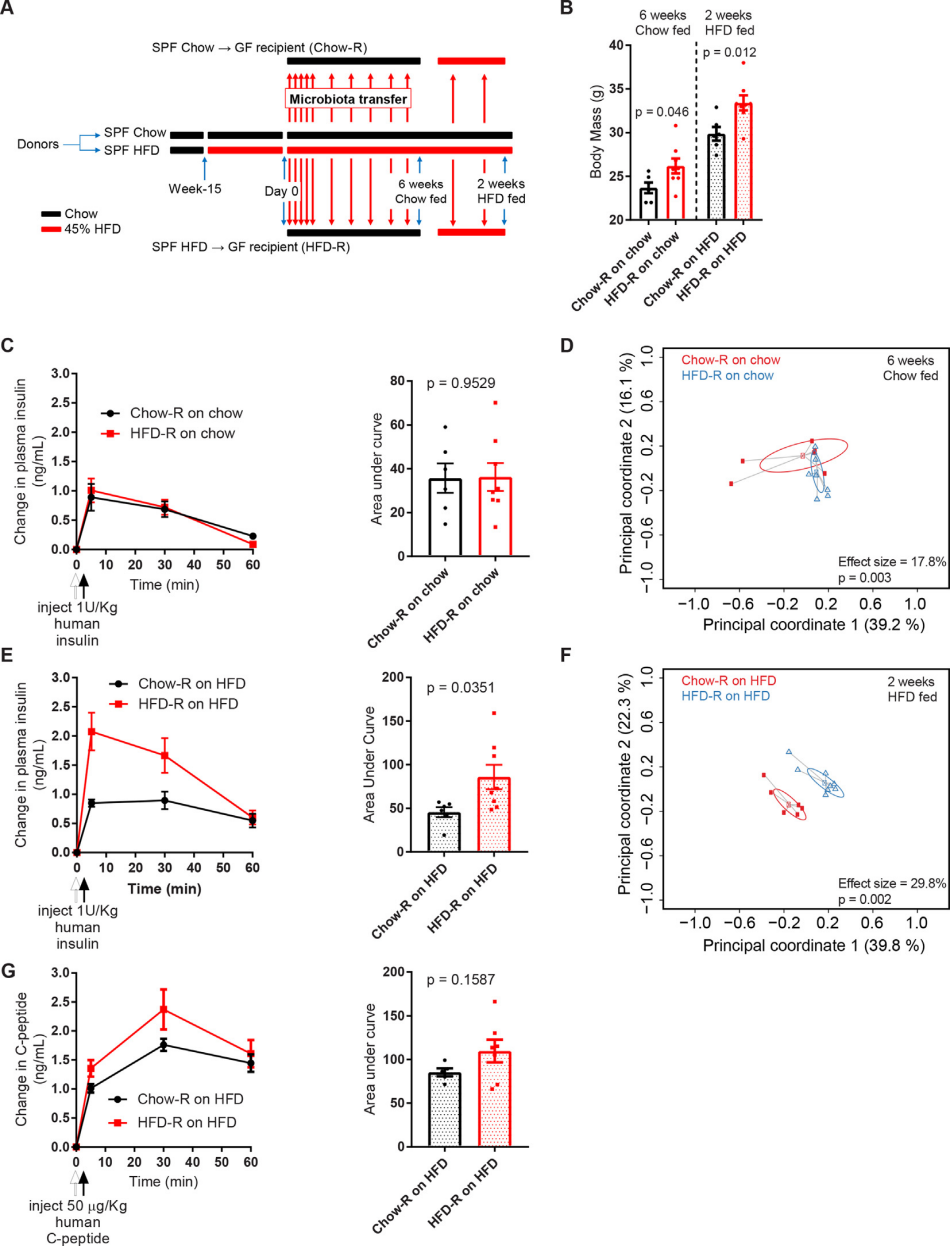
**Gut microbiota from HFD-fed mice was an independent and transmissible factor for impaired insulin clearance. A)** Schematic of the experimental design in which specific pathogen-free (SPF) donor mice were placed on a control chow diet or an obesogenic low-fiber HFD for 15 weeks. On day 0 and each subsequent day, germ-free mice (recipients) were colonized with microbiota from the donor mice for 7 days and then microbial colonization was reinforced once per week for 8–10 weeks. All of the germ-free recipient mice were tested after being on a chow diet for 6 weeks. Subsequently, all of the germ-free recipient mice were switched from a chow diet to an HFD for 2 additional weeks and retested. **B)** Body mass of germ-free recipient mice colonized with microbiota from chow diet-fed mice (Chow-R) or HFD-fed mice (HFD-R). The recipient mice were assessed after 6 weeks on a chow diet or after 2 weeks of HFD feeding (N = 6–8). **C)** Insulin clearance after injection of human insulin (1 U/kg, i.p.) was measured in germ-free recipient mice after 6 weeks of colonization, in which all of the recipient mice were fed a chow diet (N = 6–8). **D)** PCoA plot of the Bray-Curtis dissimilarity of fecal microbiota from germ-free recipient mice at the time of the insulin clearance test in 4C. **E)** Insulin clearance after injection of human insulin (1 U/kg, i.p.) measured in the same germ-free recipient mice as in 4C but after 2 weeks on an HFD. **F)** PCoA plot of the Bray-Curtis dissimilarity of fecal microbiota from the germ-free recipient mice at the time of the insulin clearance test in (E). **G)** C-peptide clearance after injection of human C-peptide (50 μg/kg) clearance in the germ-free recipient mice after 6 weeks of colonization on a chow diet plus an additional 2 weeks on an HFD (N = 5–7). **Data information:** All of the values are mean +/− SEM. Statistical significance was measured as p < 0.05 using student's t-test. Each dot/symbol indicates one mouse. The variance in the microbiome was partitioned with an Adonis analysis of variance on Bray-Curtis dissimilarities.

To investigate a causative role of the intestinal microbiota in regulating insulin clearance, we colonized germ-free mice (that is, recipient mice) with microbiota from conventional donor mice fed either a chow or an HFD containing 45% of calories from fat (Figure 4A). The donor mice were split into chow-fed or HFD-fed groups for 15 weeks prior to colonization of germ-free mice using soiled bedding from donor cages. One group of germ-free recipient mice was colonized from chow donors (Chow-R), and the other group of germ-free recipient mice was colonized from HFD donors (HFD-R). All of the mice were singly housed and fed a chow diet. Following 6 weeks of continual colonization, the germ-free recipient mice were challenged with human insulin (1 U/kg) and tested for insulin clearance (6 weeks of a chow-fed test). The HFD-R mice had higher body masses than the Chow-R mice (Figure 4B). However, there was no difference in insulin clearance between the Chow-R and HFD-R mice (Figure 4C).

There was a small but significant difference in the microbial composition between the Chow-R and HFD-R mice fed a chow diet as measured by the Bray-Curtis dissimilarity index (Figure 4D). This difference was not immediately apparent based on the separation of the Chow-R and HFD-R groups in the PCoA plots; however, Adonis analysis revealed a 17.8% effect size (p < 0.01). In comparison, the donor chow-fed mice versus donor chow-fed HFD mice formed distinct clusters with an effect size of 64% (p < 0.01, Supplemental Figure 2). We previously used this 6-week colonization protocol to transfer intestinal microbiota from obese mice to germ-free recipient mice to transfer defects in glucose metabolism, but in a previous study, donor mice were fed a more obesogenic HFD that contained 60% of calories from fat [19]. The current experiment used donor mice fed an HFD with 45% of calories derived from fat, which could explain why there was a small effect size and no difference in insulin clearance. It was also possible that the subtle differences in the microbiota between the Chow-R and HFD-R groups predisposed the HFD-R mice to defective insulin clearance, but dietary stress in the recent mice was required to unmask the phenotype. To test this hypothesis, we switched all of the recipient mice from a chow diet to an HFD for 2 weeks and repeated the human insulin (1 U/kg) challenge (2-week HFD-fed test). Based on the data in Figure 2B, which show that 2 weeks of HFD feeding was insufficient to cause defective insulin clearance, we predicted that this intervention should not alter insulin clearance unless an underlying microbial phenotype was present. The Chow-R group displayed an almost identical insulin clearance rate after 2 weeks on an HFD compared to chow diet feeding (Figure 4E), consistent with the data in Figure 2B. However, after 2 weeks of HFD feeding, the HFD-R mice displayed impaired insulin clearance relative to the Chow-R group (Figure 4E). These data suggest that the microbes in the HFD-R mice predisposed these mice toward defective insulin clearance, which manifested upon the stress of HFD feeding. Importantly, this transmissible microbiota effect on insulin clearance was unlikely due to differences in body mass between the Chow-R and HFD-R mice, as these groups differed in body mass both on the chow diet and HFD (Figure 4B). A β-diversity analysis confirmed that the microbial populations in the Chow-R and HFD-R mice were different after 2 weeks of HFD feeding (Figure 4F). Not only were the 2 recipient groups separated in the PCoA plot, but the effect size increased to 29.8% (p < 0.01). Thus, the stress of HFD feeding revealed differences in the composition of microbiota in the Chow-R versus HFD-R mice, which resulted in defective insulin clearance in the HFD-R mice. We also tested C-peptide clearance in the colonized germ-free Chow-R and HFD-R mice after 2 weeks of HFD using human C-peptide injection (50 μg/kg, i.p.). C-peptide clearance was not altered in the HFD-R relative to Chow-R mice, demonstrating that the microbiota in the HFD-fed mice did not alter clearance of a protein co-secreted with insulin (Figure 4G). Our findings show that the fecal microbiota in the diet-induced obese mice impaired insulin clearance through synergy between the microbial and dietary factors without changing their body mass.

### 3.5 HFD-induced dysbiosis was associated with defective insulin clearance

**Figure 5:**
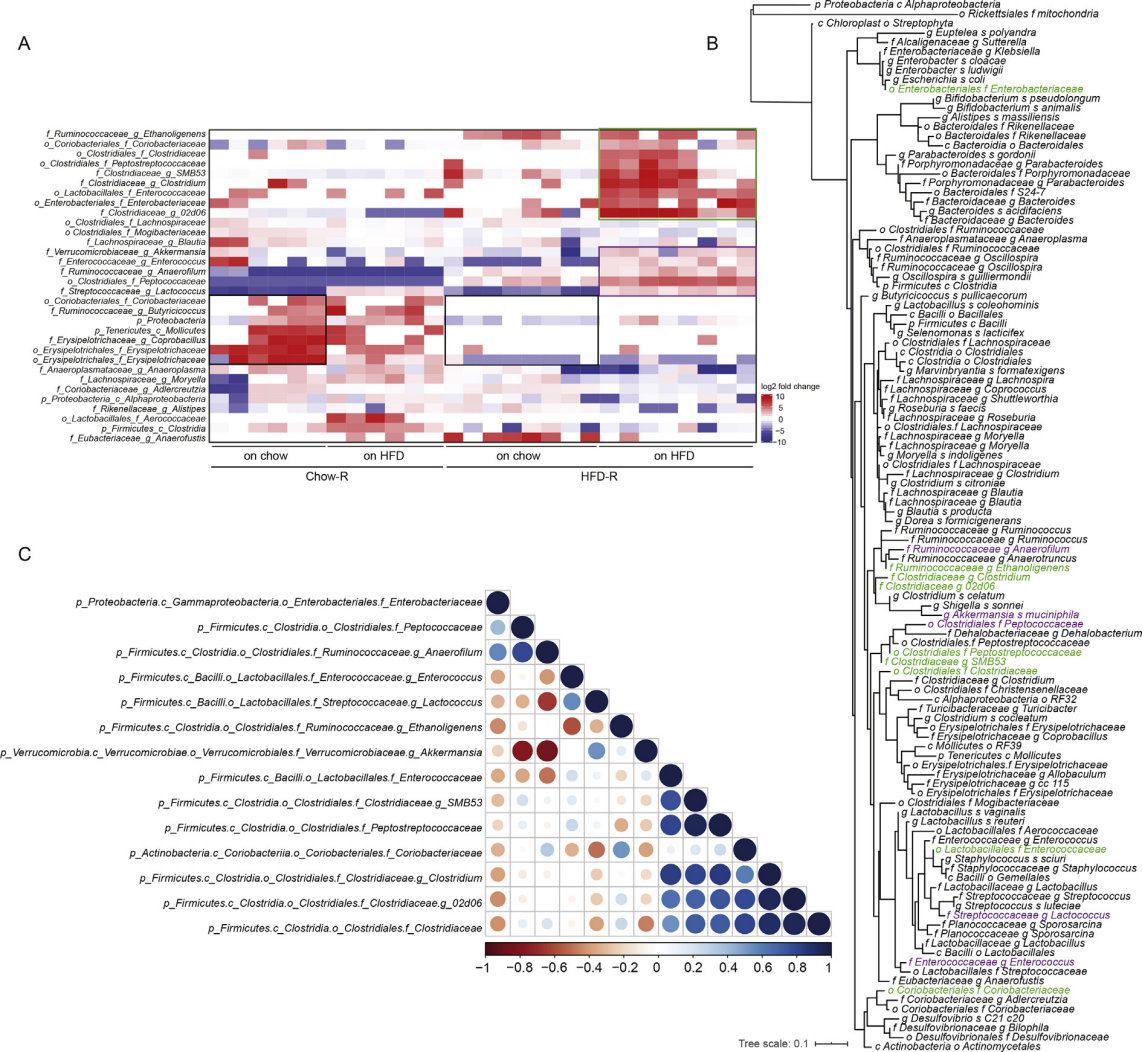
**A cluster of phylogenetically related microbes correlated with impaired insulin clearance. A)** Clustered heat map of the 32 microbial taxa that were significantly different between the Chow-R and HFD-R germ-free recipient mice (28 samples). A pairwise comparison was made between the Chow-R and HFD-R groups after 6 weeks on a chow diet and then again after 2 weeks of HFD feeding. Fold change in the relative abundance of each taxon was calculated relative to the median level across the 28 samples and plotted in the heat map. Three taxa clusters were identified based on differences between the Chow-R and HFD-R groups (cluster 1 = 2 black boxes, cluster 2 = purple box, cluster 3 = green box on the heat map). Statistical analysis was conducted using the pairwise Wilcoxon rank-sum test. Correction of multiple hypothesis testing (FDR) was calculated using the Benjamini-Hochberg method. Statistical significance was accepted at p < 0.05. **B)**Phylogenetic tree of the 16S rRNA genomic sequences of all of the taxa detected at a minimum of 10 reads in all of the recipient germ-free recipient mice. Bacterial taxa from clusters 2 and 3 are highlighted in purple and green, respectively. **C)** Pearson's co-correlation analysis of the taxa from clusters 2 and 3 of the heat map in panel A to identify pairs that are closely correlated in their relative abundance.

To identify microbial taxa that correlate with impaired insulin clearance during diet-induced obesity, we compared the fecal microbiome characteristics in the Chow-R and HFD-R mice after 6 weeks on a chow diet (Figure 4D) and after 2 weeks of HFD feeding (Figure 4F). Using a pairwise Wilcoxon test of all of the taxa (collapsed to the genus level), we compared the Chow-R to HFD-R mice at each time point separately. We hypothesized that select taxa would be uniquely changed in the HFD-R mice after 2 weeks of high-fat feeding and that these taxa would be key microbial predictors of impaired insulin clearance. The relative abundances of 32 operational taxonomic units (OTUs) significantly differed between the Chow-R and HFD-R mice at either 6 weeks on a chow diet or after 2 weeks of HFD feeding (Figure 5A).

Three clusters of taxa were delineated in the feces of the Chow-R and HFD-R mice. First, a cluster of 7 taxa showed lower relative abundance in the HFD-R mice versus Chow-R mice when both groups of mice were fed a chow diet (see Figures 5A, 2 black boxes on the heat map). These lower abundances persisted when both recipient groups were switched to an HFD. Since the HFD-R mice did not display defective insulin clearance when fed a chow diet (Figure 4C), these differences were unlikely to account for the changes in insulin clearance. The second cluster showed a group of 5 taxa whose relative abundances only increased when the HFD-R mice were fed an HFD (see Figure 5A, the purple box on the heat map). These taxa showed small to moderate differences between the Chow-R and HFD-R mice fed a chow diet, but these taxa clearly increased in the HFD-R mice fed an HFD. The third cluster showed the most dramatic shift in relative abundances related to defective insulin clearance (see Figure 5A, the green box on the heat map). These 9 taxa were very low or below detectable levels in the Chow-R group irrespective of diet. In the HFD-R group, these taxa were low or absent when the mice were fed a chow diet, but these 9 taxa significantly increased in relative abundance when the HFD-R mice were switched to an HFD, which is a condition that coincides with impaired insulin clearance.

To understand the phylogenetic relationship between the taxa identified in these clusters, we created a phylogenetic tree of the 16S rRNA genomic sequences for all of the taxa detected in the recipient germ-free mice (Figure 5B and the enlarged circular dendrogram in Supplemental Figure 3). Most taxa from clusters 2 and 3 highlighted in purple and green, respectively, were closely phylogenetically related. Four members of cluster 3 were from the *Clostridiaceae* family, and 6 of its 9 members clustered tightly. One member of cluster 3 from the *Enterococcaceae* family was similar to two members of cluster 2. Of the 14 taxa highlighted in clusters 2 and 3, only two members from the *Enterobacteriaceae* and *Coriobacteriaceae* families were located on more distal nodes. Notably, no bacterial taxa significantly decreased with the emergence of the insulin clearance defect. Changes in taxa associated with cluster 1 were not good candidates in the search for microbes that could drive defective insulin clearance, as the levels of these taxa were comparable between the chow-fed and HFD-fed conditions within each germ-free recipient group. However, microbes identified in clusters 2 and 3, which were phylogenetically similar, were potential candidates for driving defective insulin clearance during diet-induced obesity.

### 3.6 A consortium of five bacterial taxa predicted changes in insulin clearance

We next conducted Pearson correlations (Supplemental Figure 4) of the relative abundance of each taxon from clusters 2 and 3 (see Figure 5A) to the AUC from the insulin clearance test shown in Figure 4E. We focused only on the germ-free mice colonized with HFD microbiota (HFD-R) after 2 weeks of HFD feeding because this was the condition that had impaired insulin clearance relative to the Chow-R mice. In this way, a positive correlation indicated that increased relative abundance occurred in concert with decreased (that is, worse) insulin clearance. Nine out of 14 taxa positively correlated with the degree of insulin clearance (Table 1), although only two of the independent correlations were statistically significant. Table 1 lists the taxa in order of appearance in Figure 5A and ranks taxa 1–14 in order of the highest to lowest correlation coefficients. The top 7 correlations were contained in the third cluster, with 4 of these taxa belonging to the *Clostridiaceae* family. These correlations suggest that the taxa contained in cluster 3 were the best candidates for driving microbe-induced defects in insulin clearance.

**Table 1:** Pearson correlations for microbial taxa versus AUC insulin clearance.

Taxa	R	P value	Rank
p_Firmicutes.c_Clostridia, o_Clostridiales.f_Ruminococaceae.g_Ethanoligenes	0.203	0.63	9
p_Actinobacteria.c_Coriobacteriia.o_Coriobacteriales.f_Coriobacteriaceae	0.38	0.352	7
p_Firmicutes.c_Clostridia.o_Clostridiales.f_Clostridiaceae	0.514	0.193	4
p_Firmicutes.c_Clostridia.o_Clostridiales.f_Peptostreptococcaceae	0.428	0.029	5
p_Firmicutes.c_Clostridia.o_Clostridiales.f_Clostridiaceae.g_SMB53	0.405	0.319	6
p_Firmicutes.c_Clostridia.o_Clostridiales.f_Clostridiaceae.g_Clostridium	0.764	0.027	2
p_Firmicutes.c_Bacilli.o_Lactobacillales.f_Enterococcacea	0.811	0.015	1
p_Proteobacteria.c_Gammaproteobacteria.o_Enterococcacea f_Enterobacteriaceae	−0.334	0.418	12
p_Firmicutes.c_Clostridia.o_Clostridiales.f_Clostridiaceae.g_02d06	0.644	0.085	3
p_Verrucomicribia.c_Verrucomicrobiae.o_Verrucomicrobiales.f_Verrucomicrobiaceae g_Akkermansia	0.292	0.483	8
p_Firmicutes.c_Bacilli.o_Lactobacillales.f_Enterococcacea.g_Enterococcus	−0.168	0.692	10
p_Firmicutes.c_Clostridia.o_Clostridiales.f_Ruminococcaceae.g_Anaerofilum	−0.427	0.291	13
p_Firmicutes.c_Clostridia.o_Clostridiales.f_Peptococcaceae	−0.601	0.115	14
p_Firmicutes.c_Bacilli.o_Lactobacillales.f_Streptococcaceae.g_Lactococcus	−0.246	0.558	11

Pearson correlations were generated between the relative abundance values of each taxon identified in clusters 2 and 3 compared to the AUC for insulin x time during the insulin clearance test in the HFD-R mice fed an HFD diet. Taxa are listed in the same order as presented in Figure 5A and ranked 1–14 based on the highest to lowest correlation coefficients.

To identify the minimum consortium of microbes that can predict impaired (that is, lower) insulin clearance, we conducted a multiple linear regression of the taxa relative abundance versus insulin clearance in the HFD-R mice after 2 weeks of HFD feeding. Input into the model was based on the taxa correlation rank from Table 1. Relative abundances of each taxon were added to the model from the highest to lowest correlate. Before input into the multiple regression model, we conducted a Pearson co-correlation analysis of the relative abundance of the 14 taxa to identify pairs that were colinear (Figure 5C). A strong positive correlation existed between the abundances of 3 members of *Clostridiaceae* (f) taxa ranked 2, 3, and 4 in Table 1. Thus, the relative abundances of these 3 taxa were averaged to create a single input into the multiple linear regression model. The model that best explained the variance in insulin clearance in the HFD-R and high HFD-fed mice contained the top 5 taxa from the Pearson correlations: *Enterococcaceae* (f), 3 members of *Clostridiaceae* (f), and *Peptostreptococcaceae* (f) (Table 2). In this case, 92% of the variance in the insulin clearance AUC was explained by these 5 taxa. Thus, a small consortium of taxa that was selectively increased in the HFD-R mice fed an HFD for 2 weeks explained the variance in the insulin clearance. These 5 candidate taxa represent the candidate microbial community that constitute an independent factor altering insulin clearance during diet-induced obesity.

**Table 2:** Multiple linear regression model of insulin clearance.

Dependent variable: AUC insulin clearance	Adjusted R^2^	P value
Independent variables		
Model 1: p_Firmicutes.c_Bacilli.o_Lactobacillales.f_Enterococcaceae	0.6004	0.0146
Model 2: average of the co-correlated clostridia (f_Clostridiaceae + g_Clostridium + g_02d06)	0.3188	0.08415
Model 3: p_Firmicutes.c_Clostridia.o_Clostridiales.f_Peptostreptococcaceae	0.04711	0.29
Multiple linear regression: model 1 and model 2	0.556	0.05665
Multiple linear regression: model 1, model 2, and model 3	0.9243	0.003

The taxa from cluster 3 with the highest correlations to insulin AUC (see Table 1) were used to generate a multiple linear regression model to explain the variance in insulin clearance. A consortium of the top 5 taxa from the Pearson correlations: *Enterococcaceae* (f), 3 members of *Clostridiaceae* (f), and *Peptostreptococcaceae* (f) explain 92% of the variance in the insulin clearance AUC.

### 3.7 Microbial-driven impaired insulin clearance was associated with lower hepatic Ceacam-1 but increased IDE in the liver and skeletal muscle

**Figure 6:**
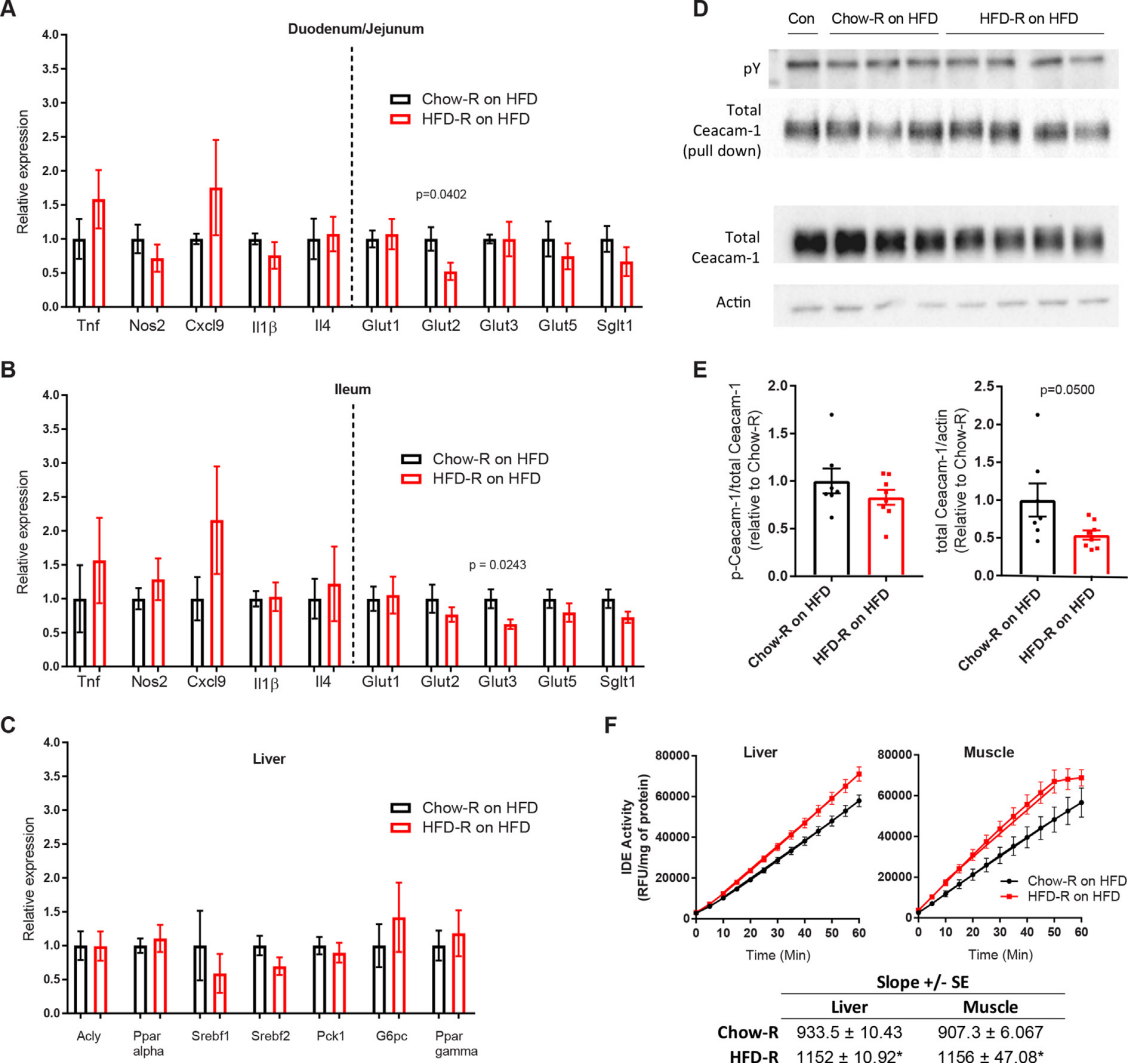
**Impaired insulin clearance was associated with lower liver Ceacam-1 and increased liver and muscle IDE activity without overt gut inflammation. A-B)** Expression of inflammatory markers and glucose transporters in the small intestine of the Chow-R mice (N = 6) and HFD-R mice (N = 7–8) fed an HFD. **C)** Expression of lipid metabolism markers in the livers of the Chow-R mice (N = 6) and HFD-R mice (N = 7–8) fed an HFD. **D)** Representative blots of tyrosine phosphorylated (pY) Ceacam-1 (top panels) and total Ceacam-1 (bottom panels) in the livers of the Chow-R and HFD-R mice fed an HFD. For pY and Ceacam-1, Ceacam-1 was immunoprecipitated and blotted with pY antibody. For the total Ceacam-1, total lysates were blotted for Ceacam-1 and compared to immunoblotting with actin. **E)** Quantification of phosphorylated and total Ceacam-1 in the livers of the Chow-R mice (N = 7) and HFD-R (N = 8) mice fed an HFD. **F)** Insulin-degrading enzyme (IDE) activity kinetics were measured in the liver and tibialis anterior muscle of the Chow-R mice (N = 7) and HFD-R (N = 8) mice fed an HFD. Linear regression was applied to activity between 10 and 50 min to calculate the reaction rates (slope). All of the values are mean +/− SEM. Statistical significance was measured as p < 0.05 using student's t-test. For A-E, the P value is stated for statistically significant differences. For F, statistical significance is marked by ∗ in the figure showing IDE activity.

We next assessed whether microbial-driven alterations in insulin clearance were accompanied by metabolic changes in the small intestine, liver, or muscle tissues of the germ-free recipient mice fed an HFD. In the duodenum/jejunum, Glut2 expression was lower in the HFD-R mice fed an HFD without changes in inflammatory markers or other glucose transporters (Figure 6A). In the ileum, Glut3 was lower in the HFD-R mice on an HFD without changes in inflammatory markers or other glucose transporters (Figure 6B). In the liver, there were no differences in the transcript levels of lipid or glucose metabolism markers in the Chow-R versus HFD-R mice fed an HFD (Figure 6C). We next assessed the tyrosine phosphorylation of Ceacam-1 and the insulin degradation pathway in the fasted Chow-R versus HFD-R mice fed an HFD. We found that tyrosine phosphorylation of Ceacam-1 relative to total Ceacam-1 was not different in the livers of the Chow-Rversus HFD-R mice fed an HFD (Figure 6D,E). However, total protein levels of Ceacam-1 were lower in the livers of the HFD-R mice fed an HFD (Figure 6D,E). Intriguingly, insulin-degrading enzyme (IDE) activity increased in both the liver and hindlimb skeletal muscle (tibialis anterior) of the HFD-R mice fed an HFD (Figure 6F). IDE catalyzes one of the final steps of insulin degradation in both the liver and muscle and an increase in IDE activity in the HFD-R mice may be a compensatory response to elevated blood insulin and a higher cumulative insulin load associated with impaired insulin clearance in the HFD-R mice.

## 4 Discussion

Our data showed that the community of intestinal microbes from the mice fed an obesogenic diet regulated their insulin clearance and that microbes could impair insulin clearance without changes in body mass. We found that a small cluster of phylogenetically related bacteria, including *Enterococcaceae*, *Clostridiaceae*, and *Peptostreptococcaceae*, explained over 90% of the variance in host insulin clearance after microbial transfer from the diet-induced obese mice. Microbe-induced changes in insulin clearance did not alter the kinetics of the C-peptide levels, indicating that the microbiota induced an effect specific to insulin rather than a co-secreted endocrine factor or general effect on peptide clearance. Our results also showed that exposure time of the host to certain microbes or microbe-derived factors was an important consideration for measuring changes in insulin clearance. Microbe-transmissible changes in insulin clearance were only evident after colonization of the germ-free mice for longer than 6 weeks followed by feeding an obesogenic diet. Neither short-term exposure to an obesogenic diet nor aging in the absence of an obesogenic diet were sufficient to alter the insulin clearance in the mice. These results demonstrated that the host must be exposed to microbes or their metabolites for a prolonged period to elicit changes in endocrine or metabolic factors, which was consistent with our previous results on the time required for microbiota to influence blood glucose control [19].

Gut microbiome composition and host genetics have been shown to alter insulin secretion. Consistent with our results, in which we found that 3 members of the *Clostridiaceae* family correlated with changes in insulin clearance, previous research that focused on insulin secretion found that *Clostridiaceae* showed the highest correlation with insulin levels [20]. It appears that changes in the relative abundance of members of the *Clostridiaceae* family are positioned to alter insulin dynamics in the host. We also found that increased *Enterococcaceae* were part of the small cluster of taxa that corelated with impaired insulin clearance. This result was consistent with changes in *Enterococcaceae* regulating insulin, since intermittent fasting lowers blood insulin and glucose and improves insulin sensitivity, coincident with a decreased relative abundance of *Enterococcaceae* in obese and diabetic db/db mice [30]. Furthermore, while bacterial LPS has been shown to impair insulin clearance, we recently demonstrated that members of the *Enterobacteriaceae* family compartmentalize in the tissues of those with T2D independently of obesity [31]. Altogether, these findings position members of the *Enterobacteriaceae* family as key players in diet-induced dysmetabolism in the host. Given the early onset of defective insulin clearance in the progression to T2D, investigating bacterial strains within *Enterobacteriaceae* that could impair insulin clearance is worthwhile.

We also found an increased relative abundance of *Peptostreptococcaceae* in the cluster of related taxa that corelated with impaired insulin secretion. Prior studies reported that feeding mice a diabetogenic HFD for at least a month increased the relative abundance of *Peptostreptococcaceae*, which was linked to lower upper gut Th17 responses, including lower RORγt CD4 T cells that provide protective immunity from diet-induced insulin resistance [32]. Intriguingly, symbiotic treatment that decreased insulin resistance and blood glucose also lowered the relative abundance of *Peptostreptococcaceae* [32]. Our data add microbe-specific regulation of insulin clearance to the growing body of evidence that specific microbes influence dynamic insulin and glucose responses and these endocrine and metabolic responses are altered by changes in the host–microbe relationship during obesity.

To the best of our knowledge, these data are the first to show that microbes from obese mice can transmit defects in insulin clearance, in which a small cluster of physiologically related microbes can account for the majority of variance in insulin clearance. There are many reports of associations between gut microbes, obesity, and insulin sensitivity or blood glucose [[33], [34], [35]]. For example, we previously showed that the relative abundance of intestinal *Clostridiaceae* was higher in mice with impaired glucose tolerance due to an obesogenic diet [19]. We also demonstrated that *Clostridiaceae* were higher in mice with microbially driven glucose intolerance and *Clostridium* was one of 9 significantly different taxa in germ-free mice colonized with microbes from HFD-fed mice [19]. This is consistent with this study's results showing elevated *Clostridium* associated with impaired insulin clearance. Prior studies identified *Prevotella copri* and *Bacteroides vulgatus* as key species in the human microbiome that can alter insulin sensitivity and intermediates such as branched-chain amino acids [36]. We have not yet identified the microbial or host metabolites that influence the mechanisms of hepatic or peripheral insulin clearance. The portal lipid load should be considered in the host-microbe regulation of insulin clearance, especially since the portal circulation connects the gut and liver, providing a direct line of communication for microbial factors and dietary metabolites. Prior research demonstrated that infusion of a dietary lipid (that is, oleate) into the portal circulation suppressed hepatic insulin clearance in dogs [37]. This is important because compared to peripheral lipid infusion, impaired insulin clearance associated with directly increasing the portal lipid load preferentially increased hepatic glucose output despite higher levels of insulin in dogs. Hence, microbial-derived lipids such as short-chain fatty acids and microbial regulation of dietary lipids in the portal circulation should be investigated as modifiers of insulin clearance. Further, these effectors should be considered in the context of insulin resistance since seminal research showed that variability in hepatic insulin clearance was a better predictor of insulin sensitivity than fasting insulin in dogs [38]. Prolonged exercise training can alter the gut microbiome in mice fed an obesogenic diet and this community of microbes in the feces after exercise training can transmit changes in the host response that increase insulin clearance without changes in adiposity [39]. Overall, there is support for a model in which impaired insulin clearance promotes transient hyperinsulinemia or prolonged periods of higher insulin load that can promote insulin resistance in a manner that is secondary to variability in body weight. In addition to exercise-induced factors, several pathways should be explored that could link microbial-host responses that modify insulin clearance, including cannabinoid receptors and adiponectin [40].

An important future goal is to define how specific microbial components alter the mechanism of tissue-specific insulin clearance. We showed that microbes were sufficient to impair insulin clearance coincident with lower total Ceacam-1 protein levels in the liver. Determining the microbial signals that can regulate liver Ceacam-1, possibly through the portal circulation, is an important future direction. One limitation was that these experiments assessed phosphorylated Ceacam-1 in fasted mice. It is an important future goal to assess how microbes alter phosphorylated Ceacam-1 during the time course of an increased post-prandial insulin load. Intriguingly, we found that microbe transmissible impairments in insulin clearance were associated with *increased* IDE activity in the liver and skeletal muscle. This was a surprising finding and raises the possibility that microbial signals engage a gut-liver and gut–muscle axis to alter tissue-resident enzymes that attempt to compensate for an increased insulin load. We found no evidence of overt intestinal inflammation, but defining the microbial molecules that alter tissue-specific insulin clearance mechanisms may involve compartmentalized immune responses. Components of the bacterial cell wall, such as LPS and muropeptides, can engage innate immune receptors in the pancreatic environment to alter insulin secretion and peripheral insulin sensitivity [16,[41], [42], [43], [44]]. It is possible that shared microbe-host responses potentiate insulin secretion and impair insulin clearance, which could increase the insulin load over time and increase the risk of complications from hyperinsulinemia, including obesity and insulin resistance.

## 5 Conclusions

We conclude that gut microbes regulate insulin clearance during diet-induced obesity. The microbes from the HFD-fed mice were contributors to impaired insulin clearance during obesity. We propose that the changes in the community of fecal microbes caused by an obesogenic diet is a stand-alone factor that can impair insulin clearance without altering C-peptide clearance or the extent of obesity. We found that gut microbes that originated from mice fed an obesogenic diet could be successfully transferred to germ-free mice and that a small cluster of these microbes predicted the majority of the variance in insulin clearance. Select members of the gut microbiota or their components and metabolites may be a target for mitigating defects in insulin clearance, which may be relevant to cumulative insulin load and the progression of obesity and type 2 diabetes.

## Author contributions

KPF researched the data, contributed to the design and discussion, and wrote the manuscript. SZ provided all of the metagenomics analysis and contributed to the discussion. BMD, NGB, FFA, JFC, BDH, CYC, MH, and TCL researched the data. JDS researched the data, derived the hypothesis, wrote the manuscript, and is the guarantor of this study.

## Data and code availability

The datasets generated during this study are available from the corresponding author on reasonable request. Figure 4, Figure 5 present associated raw data. The custom R scripts used for the data analysis are available from the corresponding author on reasonable request.

## Appendix A Supplementary data

Multimedia component 1 **Supplemental Figure 1: Incremental changes in blood insulin during an oral glucose challenge in obese mice.** Male mice were fed a control (chow) diet or an obesogenic low fiber HFD for 14 weeks. A subset of HFD-fed mice was given antibiotics (1 g/L of ampicillin and 0.5 g/L of neomycin) in their drinking water during the last 2 weeks (Chow = 17, HFD = 27, and HFD + Ab = 22). The incremental changes in blood insulin are shown to account for differences in baseline fasting insulin. **Data information:** All of the values are mean +/− SEM. Statistical significance was measured as p < 0.05 using one-way ANOVA. Post hoc analysis was conducted using Tukey's multiple comparisons test. Groups of mice denoted by different letters are statistically different from one another. Each dot/symbol indicates one mouse. Multimedia component 1

Multimedia component 2 **Supplemental Figure 2: The fecal microbiota of Chow and HFD donor mice clustered separately by the Bray-Curtis dissimilarity index.** The fecal microbiota of the chow-fed (N = 8) and HFD-fed (N = 10) donor mice were compared by the Bray-Curtis dissimilarity index. The variance in the microbiome was partitioned using an Adonis analysis of variance and significance was accepted as p < 0.05. Multimedia component 2

Multimedia component 3 **Supplemental Figure 3: Phylogenetic relationship between microbes detected in germ-free recipient mice.** An enlarged circular dendrogram of the data in Figure 5B. Multimedia component 3

Multimedia component 4 **Supplemental Figure 4: Correlations between taxa abundance and insulin clearance.** Pearson correlations were generated between the relative abundance values of each taxon identified in clusters 2 and 3 (x axis) to the insulin AUC during the insulin clearance test in the HFD-R mice on an HFD (y axis). The rank order of the highest to lowest correlation coefficients is indicated in the top right corner of each plot. Multimedia component 4
