# Supplementary figures and images for: Gut microbiota impairs insulin clearance in obese mice
Source: Mol Metab. 2020 Aug 26;42:101067. doi: 10.1016/j.molmet.2020.101067 (PMC7522491; doi:10.1016/j.molmet.2020.101067)

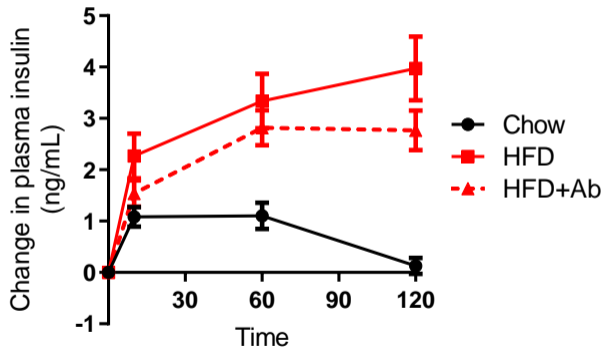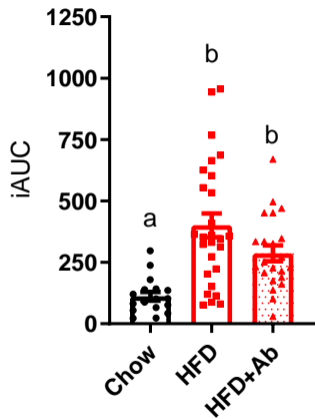

Figure S1

Supplement: Multimedia component 1 — Supplemental Figure 1: Incremental changes in blood insulin during an oral glucose challenge in obese mice. Male mice were fed a control (chow) diet or an obesogenic low fiber HFD for 14 weeks. A subset of HFD-fed mice was given antibiotics (1 g/L of ampicillin and 0.5 g/L of neomycin) in their drinking water during the last 2 weeks (Chow = 17, HFD = 27, and HFD + Ab = 22). The incremental changes in blood insulin are shown to account for differences in baseline fasting insulin. Data information: All of the values are mean +/− SEM. Statistical significance was measured as p < 0.05 using one-way ANOVA. Post hoc analysis was conducted using Tukey's multiple comparisons test. Groups of mice denoted by different letters are statistically different from one another. Each dot/symbol indicates one mouse. [file mmc1.pdf]

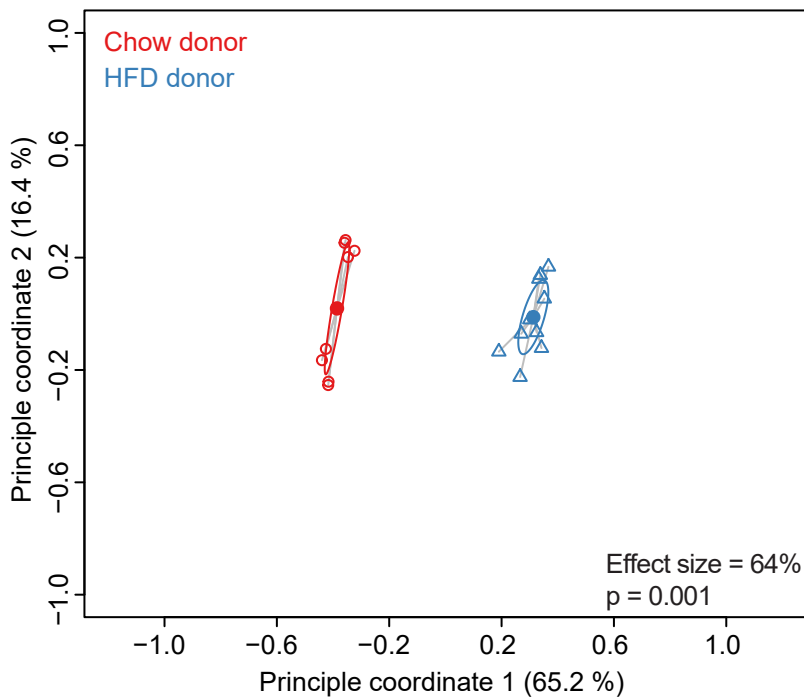

Figure S2

Supplement: Multimedia component 2 — Supplemental Figure 2: The fecal microbiota of Chow and HFD donor mice clustered separately by the Bray-Curtis dissimilarity index. The fecal microbiota of the chow-fed (N = 8) and HFD-fed (N = 10) donor mice were compared by the Bray-Curtis dissimilarity index. The variance in the microbiome was partitioned using an Adonis analysis of variance and significance was accepted as p < 0.05. [file mmc2.pdf]

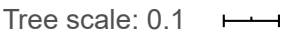

Figure S3

Supplement: Multimedia component 3 — Supplemental Figure 3: Phylogenetic relationship between microbes detected in germ-free recipient mice. An enlarged circular dendrogram of the data in Figure 5B. [file mmc3.pdf]

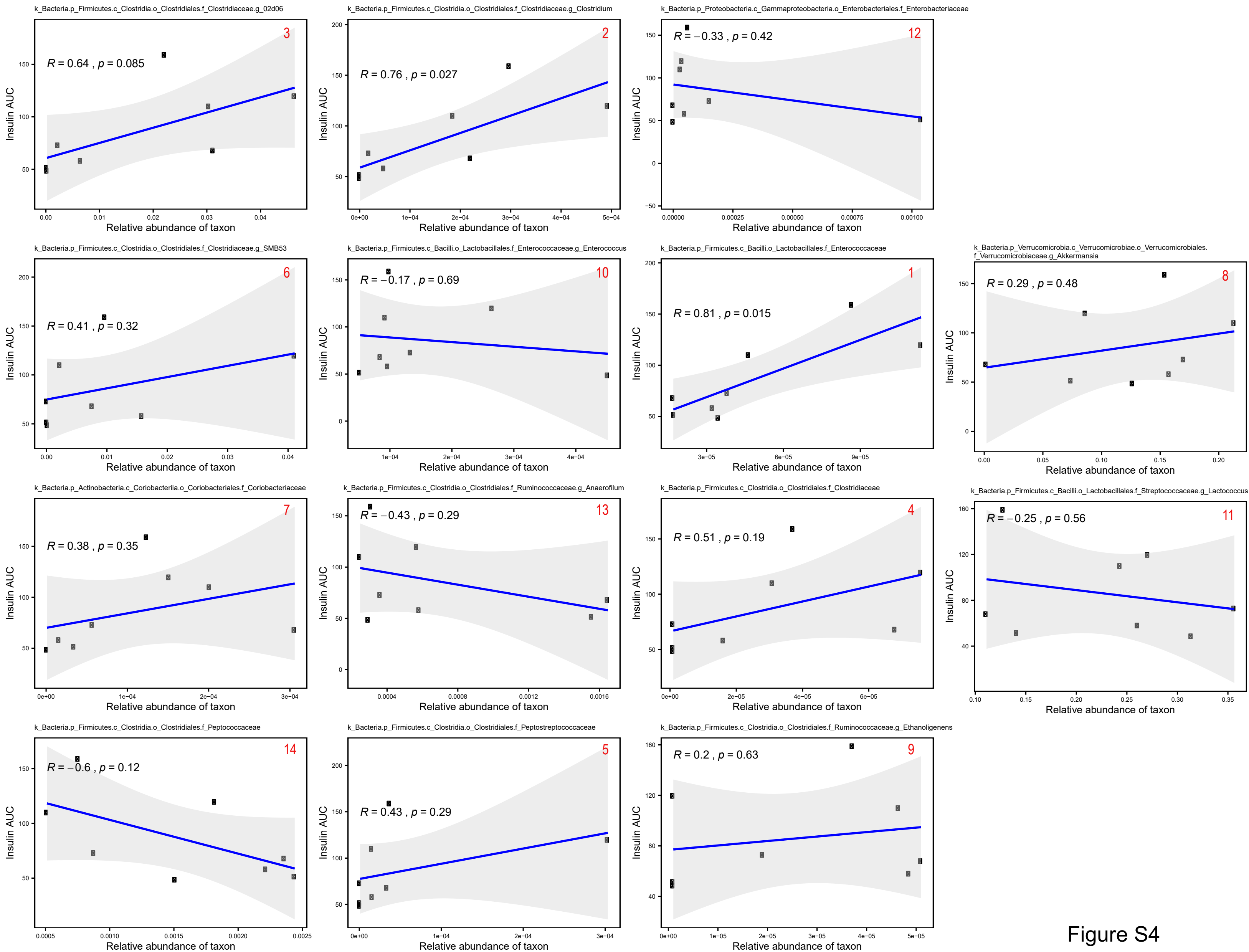

Figure S4

Supplement: Multimedia component 4 — Supplemental Figure 4: Correlations between taxa abundance and insulin clearance. Pearson correlations were generated between the relative abundance values of each taxon identified in clusters 2 and 3 (x axis) to the insulin AUC during the insulin clearance test in the HFD-R mice on an HFD (y axis). The rank order of the highest to lowest correlation coefficients is indicated in the top right corner of each plot. [file mmc4.pdf]
